# The MDM2 inhibitor CGM097 combined with the BET inhibitor OTX015 induces cell death and inhibits tumor growth in models of neuroblastoma

**DOI:** 10.1002/cam4.3407

**Published:** 2020-10-09

**Authors:** Tyler Maser, Joseph Zagorski, Shannon Kelly, Anna Ostrander, Austin Goodyke, Abhinav Nagulapally, Jeffrey Bond, Yeonhee Park, Giselle Saulnier Sholler

**Affiliations:** ^1^ Pediatric Oncology Translational Research Program Helen DeVos Children’s Hospital Grand Rapids MI USA; ^2^ College of Human Medicine Michigan State University Grand Rapids MI USA; ^3^ Public Health Sciences Medical University of South Carolina Charleston SC USA

**Keywords:** BET, MDM2, MYC, MYCN, neuroblastoma, p53

## Abstract

**Background:**

Neuroblastoma (NB) is the most common extracranial solid tumor in infants and children, with amplification of the oncogene MYCN being a hallmark of high‐risk disease and poor prognosis. Although less frequent, overexpression of MYC is similarly an indicator of poor prognosis. Most NB tumors initially respond to chemotherapy, however, most will relapse, resulting in chemoresistant disease. After relapse, there is growing evidence of p53 inactivation. MYC/MYCN and MDM2 have been shown to interact and contribute to NB growth and disease progression. MDM2 inhibitors and Bromodomain and Extra‐Terminal domain (BET) inhibitors have both shown promise in treating NB by increasing the expression of p53 and decreasing MYC/MYCN expression, respectively. Our study focuses on the combined treatment of a MDM2 inhibitor (CGM097) with a BET inhibitor (OTX015) in neuroblastoma.

**Methods:**

Two p53 wild‐type and two p53 mutant established neuroblastoma cells lines were used to test this combination. Ray design assays were used to test whether this combination was synergistically cytotoxic to NB cells. Western blots were performed to check signaling pathways of interest after drug treatment. IncuCyte imaging and flow cytometry were utilized to quantify the apoptotic and cytostatic effects of these drugs on NB cells. In vivo studies were carried out to test the antitumor effect of this combination in a living host.

**Results:**

The combination of CGM097 and OTX015 resulted in p53 activation, decreased expression of MYC family proteins and a subsequent synergistic increase in NB cell death.

**Conclusion:**

This study warrants further investigation into the combination of MDM2 inhibitors and BET inhibitors for the treatment in NB.

## Graphical abstract

Recent literature suggests that targeting MYC family genes as well as MDM2, the negative regulator of p53, may be a viable approach to the treatment of NB. We targeted these endpoints using the BET inhibitor OTX015 and the MDM2 inhibitor CGM097 and demonstrated a synergistic increase in cell death, activation of the p53 pathway, and a significant increase in time to event in vivo, further implicating these pathways in the biology and treatment of NB.

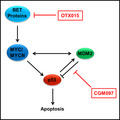

## 1 INTRODUCTION

Neuroblastoma (NB) is a cancer of the sympathetic nervous system that almost exclusively presents itself in children and accounts for roughly 15% of all pediatric cancer related deaths.^1^, ^2^ Patients with NB have a 5‐year survival rate of 74%, however, this number changes significantly depending on the classification of disease.^1^ For patients with low‐risk NB, surgery is the up‐front treatment option, with 5‐year event‐free survival rates being greater than 85%.^1^, ^2^, ^3^, ^4^, ^5^ In contrast, children with high‐risk disease respond to treatment poorly, as evidenced by the 5‐year event‐free survival rates being below 50%.^1^ High‐risk NB is often characterized by amplification of the oncogene *MYCN*, which accounts for approximately 30%‐40% of all high‐risk NB cases.^6^ Another *MYC* family member, *C‐MYC*, has also been shown to be an indicator of poor prognosis in NB and is aberrantly expressed in 10%‐11% of high‐risk NB cases.^7^, ^8^ Due to the poor outcome of high‐risk NB patients, new treatment options are needed for this cohort.

Bromodomain and Extra‐terminal domain (BET) family proteins, including BRD2, BRD3 and BRD4, recognize acetylated lysine residues on histones, which is often indicative of an open chromatin structure and active transcription.^9^, ^10^, ^11^ After recognition of acetylated lysine residues BET proteins recruit transcription factors which ultimately induce gene transcription.^10^, ^11^ BET proteins have been shown to promote the function of super enhancers which are often found upstream of cell‐specific oncogenes, including *MYCN* in NB.^12^ Previous studies have demonstrated that inhibiting BET proteins results in lower protein expression levels of both MYCN and C‐MYC, which lead to a decrease in cell viability and increase in cell cycle arrest.^13^, ^14^ Specifically, the BET inhibitor OTX015 has shown great efficacy in binding to and competitively inhibiting BRD2, BRD3, and BRD4, leading to neuroblastoma cell death in‐vitro and decreased tumor volume in vivo.^15^, ^16^ Since BET inhibitors exhibit their effects through super enhancer inhibition there are global epigenetic changes associated that are associated with this treatment. Specifically in NB cells, evidence shows that *MYC* family genes are significantly affected with BET inhibition, suggesting this mechanism may be at least partly responsible for the efficacy of these compounds in this disease.^16^ This previous work suggests that a reduction in *MYC* family protein expression, via the inhibition of BET family proteins, could be a viable option for treating high‐risk NB patients with high expression of these oncogenes.

Many adult cancers are driven by a mutation of the key tumor suppressor gene *TP53*, however, this gene is only mutated in roughly 2% of initial NB tumors.^17^ Initially, diagnosed NB tumors typically respond to chemotherapy but then stop responding when the patient relapses.^17^ In relapsed neuroblastoma the tumor suppressor protein p53 has been shown to be non‐functional in about 50% of patients but only mutated in 15% of cases.^18^ This suggests that p53 inactivation plays a major role in the ability to treat NB with chemotherapeutics. Mouse double minute 2 homolog (MDM2) facilitates p53 protein degradation by the proteasome in addition to sequestration of p53 within the cytoplasm, which renders the protein functionally inactive.^19^ Additionally, p53 induces the expression of MDM2 in an auto‐regulatory negative feedback loop.^18^, ^19^, ^20^ Dysregulation of MDM2 is common in NB and is known to play a role in suppressing p53 activity, further implicating p53 dysregulation in relapsed NB disease.^20^ Recent studies have demonstrated that the use of MDM2 inhibitors to interrupt the interaction of MDM2 with p53 is a viable experimental approach, leading to increases in cell cycle arrest and cell death.^21^, ^22^

The literature demonstrates that MDM2 can directly interact with the MYC family members MYCN and C‐MYC to stabilize one another and contribute to NB disease progression.^23^, ^24^ Experimentally, the most sensitive NB cells to MDM2 inhibition have been shown to have MYCN amplification, suggesting that MYCN may play a prominent role in the MDM2/p53 axis.^21^, ^25^, ^26^ Additionally, activation of p53 in the presence of abundant MYCN leads to nuclear co‐localization of the two proteins which alters the p53 stress response, further intertwining these pathways in NB.^27^ Taken together, MDM2 dysregulation in NB and the role of MYC family proteins in high risk disease has led us to hypothesize that targeting both the MDM2 and MYC family pathways simultaneously may result in a synergistic increase in cytotoxicity in NB models, and present a possible novel therapeutic option.

## 2 MATERIALS & METHODS

### 2.1 Drugs

The MDM2 inhibitor CGM097 was donated by Novartis (Basel, Switzerland). The BET inhibitor OTX015 was purchased from ApexBio (A3692, Boston, MA). Both drugs were diluted to a stock concentration of 10 mM in DMSO. Stock of both drugs was sub‐aliquoted into small single‐use volumes to prevent freeze‐thaw cycles and were appropriately stored at −20°C. Drugs were diluted in pre‐warmed media to their desired concentration immediately prior to use.

### 2.2 RNA expression profiling

5 × 10^4^ cells were harvested and centrifuged at 300 g for 5 minutes to pellet the cells. Cells were washed with 500 µL PBS (10010‐049, Thermo Fisher Scientific, Waltham, MA) and centrifuged at 300 g. Cells were washed with 500 µL of PBS again with the addition of 1 µL of SuperaseIN RNase inhibitor (AM2694, Thermo Fisher Scientific) and centrifuged at 500 g. This process was repeated once. Cells were then washed with PBS a final time with a volume of 250 µL with the addition of 2 µL of SuperaseIN RNase inhibitor and centrifuged at 500 g. Samples were frozen and stored at −80°C until they were shipped to the Clinical Research Laboratory (Lenexa, KS) where the Affymetrix GeneChip U133 Plus 2.0 genome wide expression cDNA microarray was used for the quantification of RNA expression. Affymetrix arrays were processed using the “affy” Bioconductor package (Release 2.13) through R (v3.0).^28^

### 2.3 Cell culture

**Figure 1:**
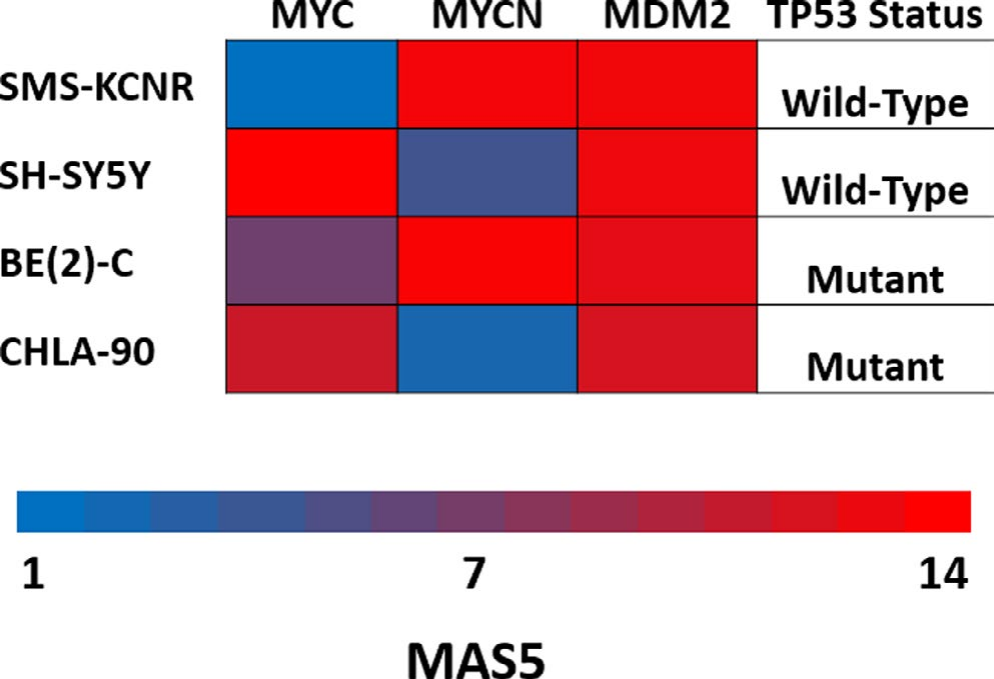
Neuroblastoma established cell line gene expression. Gene expression data for genes of interest in all cell lines used throughout this manuscript. Gene expression data are based upon MAS5 scores

The following neuroblastoma cell lines were used in the preparation of this manuscript: SMS‐KCNR (donated by Dr John Maris in 2004, The Children's Hospital of Philadelphia, PA, available by name from Childhood Cancer Repository, Lubbock, TX), SH‐SY5Y (CRL‐226, ATCC, Manassas, VA), BE(2)‐C (CRL‐2268, ATCC), and CHLA‐90 (donated by Dr Patrick Reynolds in 2006, The Children's Hospital of Los Angeles, CA, available by name from Childhood Cancer Repository, Lubbock, TX). SMS‐KCNR and SH‐SY5Y cell lines were used in this manuscript due to their wild‐type p53 and high expression of MYC genes (Figure 1). BE(2)‐C and CHLA‐90 cell lines were used in this manuscript due to their mutant p53 and high expression of MYC genes (Figure 1). All cell lines were cultured under the same conditions: RPMI 1640 media (11875‐119, Thermo Fisher Scientific, Waltham, MA) supplemented with 10% fetal bovine serum (A3160502, Thermo Fisher Scientific) and 1% penicillin‐streptomycin (15140163, Thermo Fisher Scientific). All experiments were conducted on cell populations with a minimum of 85% viable cells as determined by Trypan Blue (SV3008401, Thermo Fisher Scientific) counts immediately prior to plating. When harvested for experiments all cells were centrifuged at 300 g unless otherwise specified. All cell lines were kept in an incubator at 37°C with 5% CO_2_. All cell lines were certified mycoplasma free and STR authenticated (IDEXX Laboratories Inc, Westbrook, ME).

### 2.4 Cell viability

Cells were plated in 96‐well clear bottom, black walled tissue culture plates (3904, Corning, Kennebunk, ME) and allowed to attach overnight in the incubating conditions specified above. Cells were treated with increasing concentrations of CGM097 or OTX015 and incubated for 48 hours. After incubation, media was removed from the cells and replaced with 2 µM/mL Calcein AM (C3100MP, Thermo Fisher Scientific) in pre‐warmed PBS. Plates were subsequently incubated at 37°C for 30 minutes. Following incubation, fluorescence was quantified from each well of the plate at 480ex/520em with the Synergy HTX Multi‐Mode Reader (BioTek Instruments, Winooski, VT). All data analyzed was normalized to vehicle control. Vehicle for all cell viability experiments was media supplemented with 0.7% DMSO. EC50 values were calculated using a four‐parameter variable‐slope dose‐response curve through the GraphPad Prism v.5 software (GraphPad Software Inc, La Jolla, CA).

### 2.5 Ray design

Drug combination studies were performed to evaluate synergism between CGM097 and OTX015 in the treatment of NB cells. Cells were plated in three 96‐well clear bottom, black‐walled plates (3904, Corning) per experiment for three technical replicates per dose. Dosing schemes for both drugs were calculated around each single‐agent EC50 value for each cell line with consistent proportional doses above and below the EC50 values. From this, six dose‐response curves (referred to as rays) were created with two curves being reserved for single‐agent alone (Rays 1 and 6). The other four dose response curves consisted of statistically determined dose proportions of both drugs combined together (Rays 2‐5). Cells were drugged with single‐agent or combinations of both drugs and allowed to incubate for 48 hours. After incubation the standard cell viability protocol and EC50 value calculation detailed above were performed. The EC50 values were calculated twice per dose response curve to obtain EC50 values for each agent with their corresponding doses. The EC50 values for each dose response curve were then plotted with the single‐agent EC50 values being the x‐ and y‐ intercepts for our line of Loewe additivity. For statistical analysis, all observations with the component doses at a fixed ray were used to fit dose‐response curves using a logistic regression model in Chou and Talalay (1984) given by

$$\log\frac{E}{1‐E}=\beta_{0,i}+\beta_{1,i}\log d_{i},i=1,2$$

and

$$\log\frac{E}{1‐E}=\beta_{0,c}+\beta_{1,c}\log{(d}_{1}+d_{2}),$$

where E denotes dose producing effect, and $d_{1}$ and $d_{2}$ denote the combination doses for each drug. Then, for a fixed effect EC50, the interaction index was estimated by.

$$\hat{\tau }=\frac{{\hat{D}}_{c}d_{1}/\left(d_{1}+d_{2}\right)}{\hat{D_{1}}}+\frac{{\hat{D}}_{c}d_{2}/\left(d_{1}+d_{2}\right)}{\hat{D_{2}}},$$

Where $\hat{D_{1}}=\exp\left(‐\frac{\hat{\beta_{0,1}}}{\hat{\beta_{1,1}}}\right),\;\hat{D_{2}}=\exp\left(‐\frac{\hat{\beta_{0,2}}}{\hat{\beta_{1,2}}}\right)$, and $\hat{D_{c}}=\exp\left(‐\frac{\hat{\beta_{0,c}}}{\hat{\beta_{1,c}}}\right).$ ^29^ Under the normality assumption on regression parameters we computed the bootstrap standard deviations of the estimate of interaction index based on 500 bootstrap samples and constructed the confidence intervals for interaction index.^30^ According to the Loewe additivity model, if the interaction index is less than 1 and the upper bound 95% confidence interval (CI) excludes 1 then the combination dose is synergistic. If the 95% CI for the interaction index includes 1 then the combination dose is additive.^31^

### 2.6 Western blot analysis

Cells were plated in six‐well tissue culture plates (3516, Corning) and allowed to attach overnight in the incubating conditions specified above. Cells were left untreated or treated with vehicle, (0.05% DMSO), 200 nM CGM097, 5 µM OTX015, or a combination of both drugs at their respective doses. After 4 hours of incubation with the drug, media was removed, plates were put on ice, and cells were washed with cold PBS (10010049, Thermo Fisher Scientific). PBS was removed and RIPA Lysis Buffer (89901, Thermo Fisher Scientific) with protease inhibitor (88665, Thermo Fisher) was added dropwise to each well, making sure to cover the entire surface area of the well. Plates were incubated on ice for 15 minutes. Cell lysate was then collected and immediately frozen at −80°C. Protein content within each sample was quantified with a Bradford assay using the Protein Assay Dye Reagent Concentrate (5000006, Bio‐Rad, Hercules, CA). A total of three separate rounds of whole cell lysate were collected from each cell line. Sample was diluted in 6× Laemmli SDS buffer (J61337, Alfa Aesar, Haverhill, MA) and equal amounts of protein (30 µg) for each sample was added to each well of a 12% SDS polyacrylamide gel, utilizing two gels per round of lysate. Samples were then electrophoresed at 100 V for 1.5 hours. Samples were then semi‐dry transferred to a 0.2 µm supported nitrocellulose membrane (1620096, Bio‐Rad) using the Trans‐Blot Turbo Transfer System (1704150, Bio‐Rad). Membranes were blocked in Tris‐Buffered Saline (1706435, Bio‐Rad) with 0.1% Tween 20 (1 706 531, Bio‐Rad) (TBST) and 5% non‐fat dry milk for 1 hour at room temperature. All primary antibodies were diluted 1:1000 in 5% milk in TBST and incubated on membranes overnight. Membranes were then washed with TBST 3 times for 5 min/wash. Secondary antibody diluted 1:2000 in 5% milk in TBST was then added to membranes and incubated for 1 hour at room temperature. Membranes were again washed for three times for 5 min/wash. Clarity Western ECL Substrate (1705061, Bio‐Rad) was applied to the membranes for 5 minutes. Membranes were then imaged using the ChemiDoc XRS System with Image Lab Software v5.2.1 (1708265, Bio‐Rad). Primary antibodies include MDM2 (86934), c‐MYC (9402), N‐Myc (9405), p53 (2527), p21 Waf1/Cip1 (2947), and β‐Actin (4967). Anti‐rabbit IgG, HRP‐linked (7074) secondary antibody was used. All antibodies were purchased from Cell Signaling Technology (Danvers, MA). Individual bands were quantified using the Image Lab Software v5.2.1 (Bio‐Rad). Bands were background reduced, normalized to loading control (β‐Actin to each respective blot) and then normalized to the untreated control. After probing for each protein of interest blots were stripped by Restore Plus Western Blot Stripping Buffer (46430, Thermo Fisher Scientific), as per the manufacturer's protocol. Statistical significance between groups was determined by a one‐way ANOVA followed by Tukey's multiple comparison test using GraphPad Prism v.5 software (GraphPad Software Inc). Bands with no expression in untreated control were normalized to background.

### 2.7 Cell cycle analysis

Cells were plated in six‐well plates tissue culture plates (3516, Corning) and allowed to adhere overnight. Cells were treated with Vehicle (0.05% DMSO), CGM097 (200 nM), OTX015 (5 µM), or a combination of both drugs at their respective doses. Twenty‐four hours after treatment, cells were harvested, fixed/permeablized, and labeled with FxCycle PI/RNAse Staining Solution (F10797, Thermo Fisher Scientific), as per the manufacturer's protocol. After labeling, cell cycle was analyzed by measuring DNA content, using a ZE5 flow cytometer (Software v2.3.03.0, Bio‐Rad). After analysis on the flow cytometer, FCS files were exported and analyzed using FlowJo software v10.5.3 (FlowJo LLC, Ashland, OR). G1 cell cycle results were tested for statistical significance with a one‐way ANOVA followed by Tukey's multiple comparison test using GraphPad Prism v.5 software (GraphPad Software Inc). The gating strategy used in the cell cycle analysis is presented in Figure S2.

### 2.8 IncuCyte imaging assay

Cells were plated in 96‐well clear bottom, blacked walled tissue culture plates (3904, Corning) and allowed to attach overnight in the incubating conditions specified above. Cells were treated with vehicle (0.05% DMSO), 200 nM CGM097, 5 µM OTX015, or a combination of both drugs at their respective doses. All treatment groups were also treated with IncuCyte Caspase‐3/7 Green Apoptosis Assay Reagent (4440, Essen Bioscience, Ann Arbor, Michigan) diluted to a final concentration of 5 µM. Plates were placed in the IncuCyte ZOOM (Essen Bioscience) and 9 images were taken with a 20× objective in every well, every 4 hours for 48 hours. Using the IncuCyte ZOOM 2016B software (Essen Bioscience) a fluorescence threshold was set on representative images and then applied to all images within the experiment allowing for unbiased quantification of fluorescence across all treatment groups and time points. Statistical significance between groups was calculated using a repeated measures ANOVA followed by Tukey's multiple comparison test using GraphPad Prism v.5 software (GraphPad Software Inc).

### 2.9 In‐vivo *studies*

Animal studies were carried out in 6‐week‐old athymic female NU(NCr)‐*Foxn1^nu^* mice (Van Andel Research Institute, Grand Rapids, MI). SMS‐KCNR cells were resuspended in Matrigel (354 234, Corning) at a concentration of 20 × 10^6^ cells/mL and 100 µL was injected subcutaneously into the right flank of 40 mice (2 × 10^6^ cells/mouse). Tumors were allowed to establish for 7 days post‐injection. Tumors were measured with a caliper to calculate tumor volume and mice were randomized into four groups (10 mice/group) with similar tumor volume averages. Mice were treated with either vehicle (10% DMSO: 90% polyethylene glycol 300), 70 mg/kg CGM097, 50 mg/kg OTX015, or a combination of both drugs at their respective doses. Drug was administered by oral gavage daily for 28 days. Mouse tumors were measured twice per week until the tumors reached 2000 mm^3^ at which point the tumors were measured three times per week. Mice were humanely euthanized by CO_2_ asphyxiation and cervical dislocation as a secondary assurance of death once tumors reached the maximum volume of 3000 mm^3^. Mice were weighed daily while on drug treatment and once weekly once treatment was stopped. If tumors ulcerated before reaching the maximum tumor volume they were censored from survival analysis. The experiment was conducted two independent times. Statistical significance in tumor volume between treatment groups was determined by the Friedman test followed by Dunn's multiple comparison test. Kaplan‐Meier plots were analyzed by the log‐rank test with the overall p‐value and individual hazard ratios between treatment groups reported. Statistical tests were conducted using GraphPad Prism v.5 software (GraphPad Software Inc) All study animals were single‐housed in Optimice cages (C79100PFF, Animal Care Systems, Centennial, CO), with 1/8” corn cob bedding, irradiated bed nests, and diamond twists with diet (8940, Envigo, Huntingdon, United Kingdom) and reverse osmosis water offered ad libitum. Mice were cared for in accordance with the Guide for the Care of and Use of Laboratory Animals adopted by the National Institutes of Health and Michigan State University Institutional Animal Care and Use Committee guidelines (IACUC Approval No. XXXXX).

### 2.10 Statistical analysis

All experiments were completed as three biological replicates with three technical replicates each, excluding the in vivo studies which were completed two individual times with ten mice per group. Individual statistical tests for each experiment can be found in the materials and methods and figure legends where applicable. All statistical tests were conducted with an α = 0.05. All data reported in this manuscript are represented as Mean ± Standard Deviation.

## 3 RESULTS

### 3.1 CGM097 and OTX015 combination treatment results in a synergistic increase in cell death

**Figure 2:**
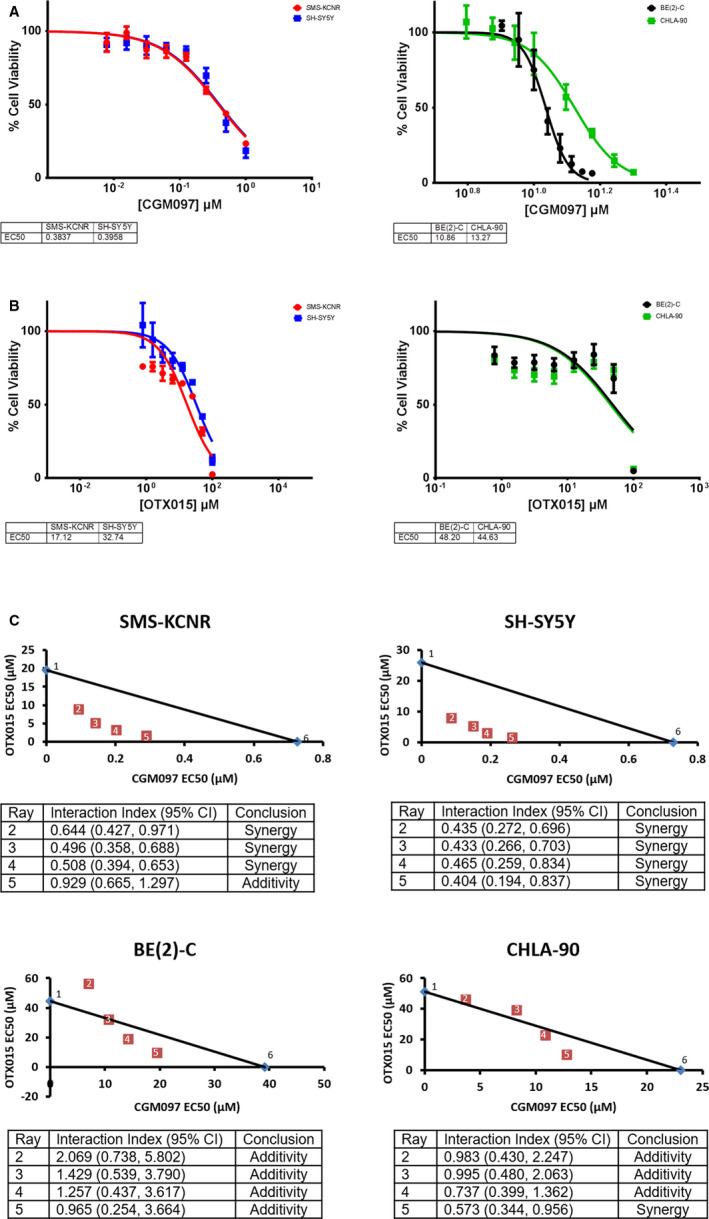
EC50 values and ray design combination assays for CGM097 and OTX015 after 48 hours of treatment. A, B, CalceinAM cell viability assay was utilized on NB cell lines treated with increasing concentrations of CGM097 and OTX015 for 48 hours to determine EC50. C, Ray designs combining different ratios of CGM097 and OTX015 were utilized to determine potential drug synergism after 48 hours of drug treatment. Graphs are representative of three independent experiments. Numbers 1‐6 on each graph represent ray numbers which correspond to the interaction index tables. Numbers 1 and 6 are reserved for the single‐agent EC50 values while numbers 2‐5 represent the combination EC50 values. Three biological replicates were conducted with three technical replicates per biological replicate. The tables identify those with a synergistic effect based upon interaction indices and 95% confidence intervals below each graph

The purpose of the present study was to determine if the MDM2 inhibitor CGM097 and BET inhibitor OTX015 would, in combination, result in a synergistic increase in NB cell death. Toward this end, EC50 values were determined using the Calcein AM fluorescent assay after 48 hours of treatment. NB cells with wild‐type p53 and high expression of MYC family oncogenes (SMS‐KCNR and SH‐SY5Y) had EC50 values ranging from 0.384‐0.396 µM and NB cells with mutated p53 and high expression of MYC family oncogenes (BE(2)‐C and CHLA90) had EC50 values ranging from 10.86‐13.17 µM, when treated with CGM097 (Figures 1 and 2A). Similarly, the NB cell lines used in this manuscript had EC50 values ranging from 17.12 to 48.20 µM when treated with OTX015 (Figure 2B).

Once EC50 values were established for all cell lines, ray design dosing schemes were statistically generated to determine if combining these drugs synergistically kills NB cells. The EC50 values for each ray were plotted on graphs with the x‐axis representing the CGM097 EC50 values and the y‐axis representing the OTX015 EC50 values (Figure 2C). A trend line was drawn from the single‐agent EC50 values to represent the line of Loewe additivity. From the dose‐response curves interaction indices and their 95% confidence intervals were calculated to statistically determine if these drugs are acting synergistically across the different treatment rays. The EC50 values from each treatment ray consistently fall below the line of Loewe additivity for SMS‐KCNR and SH‐SY5Y cells, with only one interaction index for one of the SMS‐KCNR rays not meeting the requirements to be deemed statistically synergistic (Figure 2C). This would suggest that CGM097 and OTX015 synergistically decrease viability in these cell lines. While some EC50 values fall below the line of Loewe additivity, there was only one treatment ray with an interaction index that was deemed statistically synergistic between BE(2)‐C and CHLA‐90, suggesting that these cell lines are more resistant to the combination of CGM097 and OTX015 (Figure 2C).

### 3.2 CGM097 and OTX015 increase p53 expression and decrease MYC family proteins, respectively

**Figure 3:**
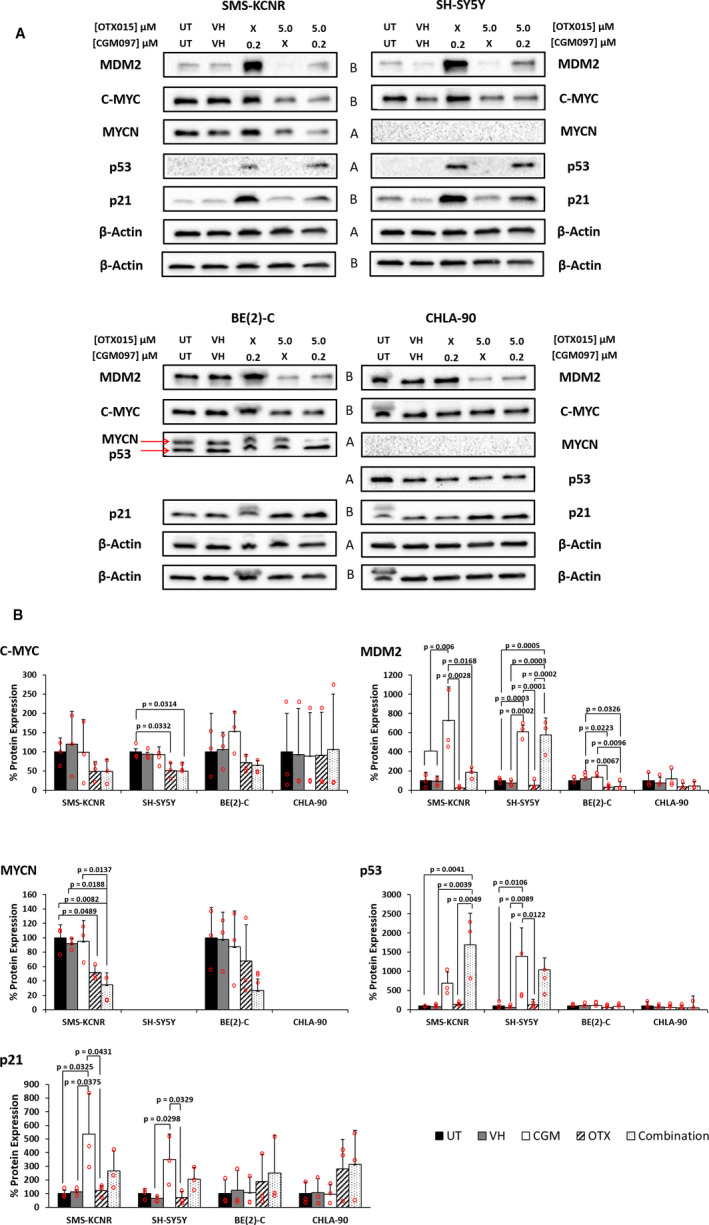
Western blot analysis shows increase in p53 protein expression and decrease in MYC family protein expression. A, SMS‐KCNR, SH‐SY5Y, BE(2)‐C, and CHLA‐90 cells were untreated, treated with vehicle, 200 nM CGM097, 5 µM OTX015, or a combination of both drugs and analyzed via western blot. Two blots were used per replicate due to conflicting molecular weights amongst targets. The letters between blots indicate which targets correspond to which β‐Actin band. B, Individual bands from three biological replicates were background reduced, normalized to their own β‐Actin control and then normalized to UT control. Data in graphs are represented as Mean ± SD. Red circles represent the result from each biological replicate performed. Data were analyzed by a one‐way ANOVA followed by Tukey's multiple comparison test. Blot images were cropped for clarity. n.d., Not detected, UT, Untreated, VH, Vehicle, and X = Drug not given in this treatment group

Protein expression for targets of interest was analyzed via western blots after NB cells were treated with CGM097 and OTX015 for 4 hours. MDM2 protein expression was significantly increased when SMS‐KCNR and SH‐SY5Y cells were treated with CGM097, however, when the same lines were treated with OTX015 alone, MDM2 expression was almost completely abolished (Figure 3). In contrast, MDM2 protein expression was not increased to the same extent with CGM097 treatment in the mutant p53 lines BE(2)‐C and CHLA‐90, but MDM2 expression did significantly decrease when treated with OTX015 or the combination in BE(2)‐C cells. (Figure 3). MYCN and C‐MYC protein expression similarly decreased with OTX015 or combination treatment, in SMS‐KCNR and SH‐SY5Y, respectively (Figure 3). Interestingly, combination treatment induced a significant increase in p53 protein expression in SMS‐KCNR cells compared to control, while the increase in p53 with single‐agent CGM097 was not significant (Figure 3). Conversely, combination treatment did not induce a significant increase in p53 protein expression in SH‐SY5Y cells compared to control, however, the single‐agent CGM097 treated cells did experience a significant increase in p53 expression (Figure 3). p21 expression increased with CGM097 treatment in both wild‐type p53 lines, SMS‐KNCR and SH‐SY5Y (Figure 3). CGM097, OTX015, nor the combination treatment had a significant effect on the expression of p53, p21, C‐MYC or MYCN in BE(2)‐C or CHLA‐90 cells (Figure 3). Uncropped western blots for the bands used in Figure 3 are presented in Figure S1.

### 3.3 OTX015 alone and in combination causes a G1 arrest in NB

**Figure 4:**
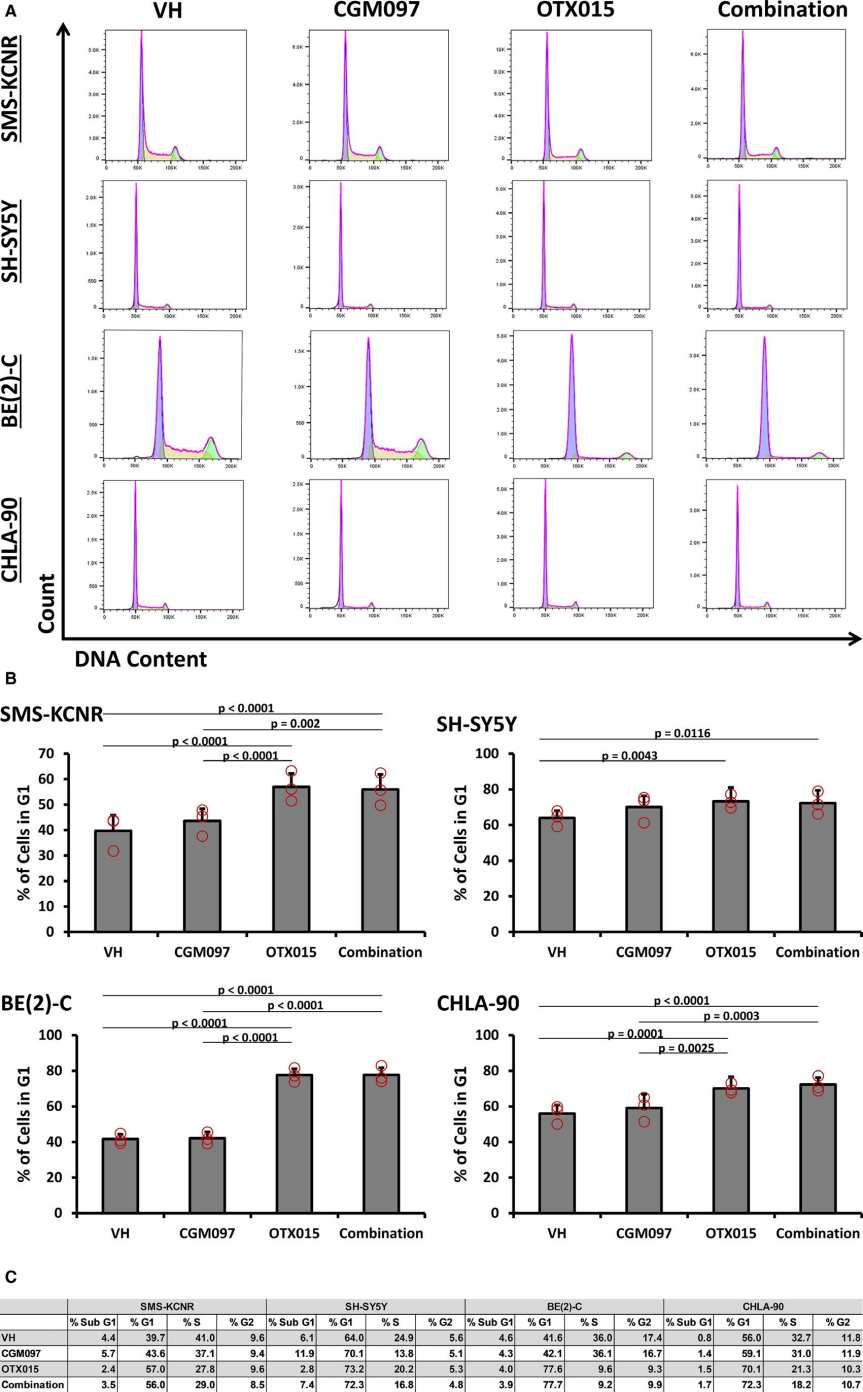
OTX015 alone and in combination induces G1 arrest in NB cells. A, Histogram plots reporting cell cycle analysis for SH‐SY5Y, BE(2)‐C, and CHLA‐90 cells after treatment with vehicle (VH), 200 nM CGM097, 5 µM OTX015, or a combination of both drugs. B, Graphic representation of the percentage of cells in the G1 phase of the cell cycle with treatment. Red circles represent the average from each biological replicate. C, Table displaying the complete cell cycle analysis for all cell lines. Cell cycle histograms are representative of three independent experiments. Three biological replicates were conducted with three technical replicates per biological replicate. Statistical comparisons were made only on G1 data. Data in graphs are represented as Mean ± SD from three independent experiments and the table lists the mean values of cell cycle percentage from all three independent experiments. Data were analyzed by a one‐way ANOVA followed by Tukey's multiple comparison test

Induction of the p53‐p21 pathway can lead to a G1 cell cycle arrest in mammalian cells.^20^ Given the induction of both p53 and p21 in SMS‐KCNR and SH‐SY5Y cells at early time points with CGM097 treatment, we next analyzed NB cell lines for cell cycle arrest using flow cytometry after 24 hours of drug treatment. SMS‐KCNR and SH‐SY5Y cells, expressing a wild‐type p53, demonstrated a significant G1 cell cycle arrest with single‐agent OTX015 increasing the number of cells in G1 by 17.3% and 9.2%, respectively, and combination treatment increasing the number of cells in G1 by 16.3% and 8.3%, respectively, compared to vehicle (Figure 4). Interestingly, single‐agent CGM097 treatment did not induce a significant G1 arrest in SMS‐KCNR or SH‐SY5Y cells, only increasing the number of cells in G1 by 3.9% and 6.1%, respectively, compared to vehicle (Figure 4). Similarly, BE(2)‐C and CHLA‐90 cells, harboring non‐functional p53 proteins, only saw a significant increase in G1 cell cycle arrest when treated with OTX015, increasing the number of cells in G1 by 36% and 14.1%, respectively, and the combination treatment, increasing the number of cells in G1 by 35.6% and 16.3%, respectively, compared to vehicle (Figure 4).

### 3.4 CGM097 and OTX015 combination treatment results in a greater induction of caspase‐3/7 activity

**Figure 5:**
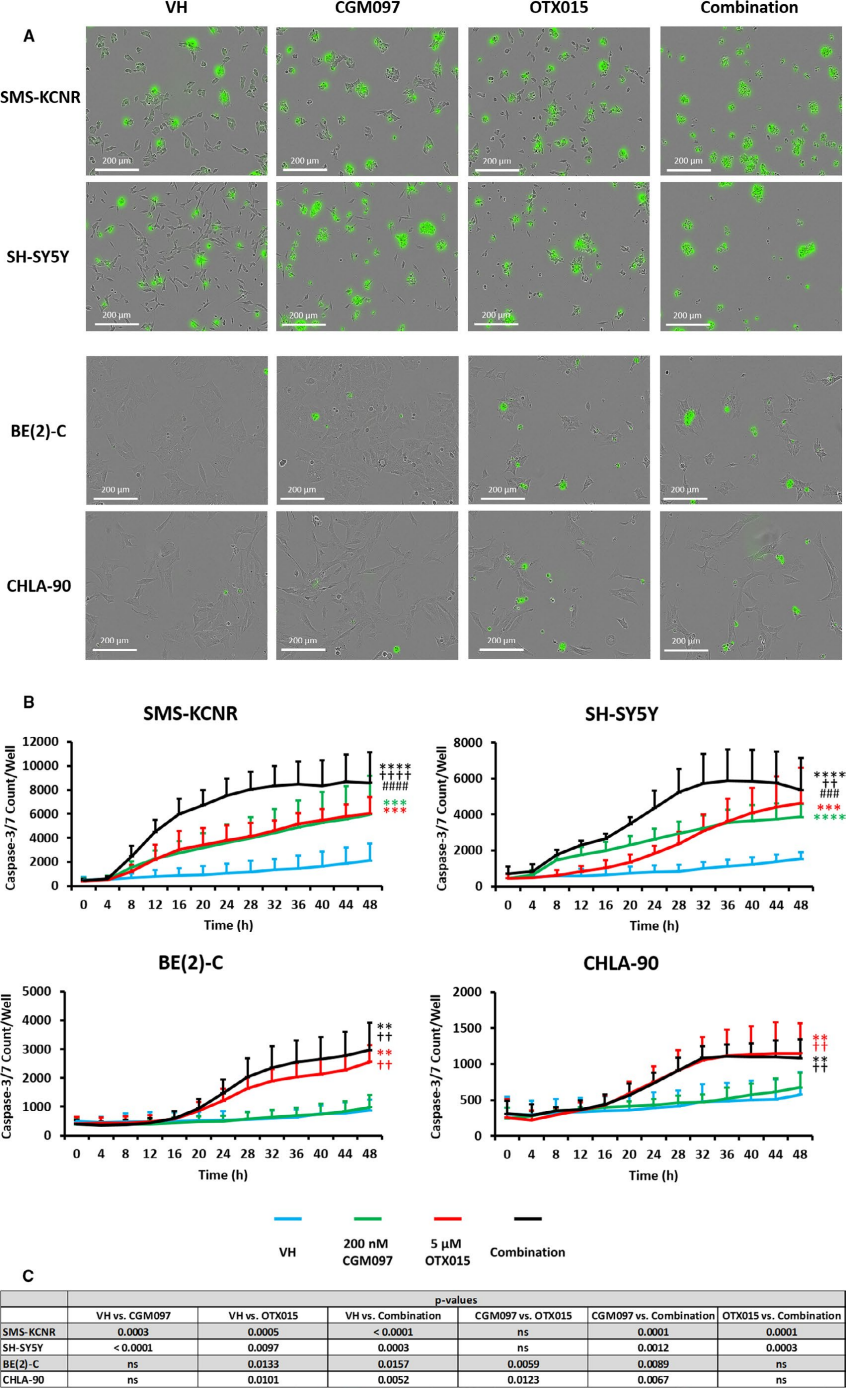
IncuCyte ZOOM analysis of caspase‐3/7 activity with single‐agent or combination treatment over time. A, Representative images of caspase‐3/7 induction at 48 hours. Images were taken with a 20× objective. B, Graphic representation of the amount of caspase‐3/7 induction per well with vehicle (VH), single‐agent (200 nM CGM097, 5 µM OTX015) or combination treatment over time. Three biological replicates were conducted with three technical replicates per biological replicate. Data are represented as Mean ± SD. C, Data were analyzed by a repeated measure ANOVA followed by Tukey's multiple comparison test. * = statistical significance vs VH, † = statistical significance vs CGM097, and # = statistical significance vs OTX015. ns, not significant

Activation of p53 is known to facilitate the induction of apoptosis, which is carried out by a cascade of caspase proteins.^20^ Therefore, we next directly measured caspase protein activity, using the IncuCyte ZOOM imaging system. Functional caspase activity was kinetically monitored as a measure of the induction of apoptosis in NB cell lines after treatment with single‐agent or a combination of CGM097 and OTX015. When treated with a combination of both drugs, SMS‐KCNR and SH‐SY5Y exhibited pronounced increases in activated caspase‐3/7, as well as a quicker induction of caspase activity, compared to those cell lines treated with single‐agent alone (Figure 5). In most cell lines caspase 3/7 activity reached its maximum threshold at 32 hours, at which point the combination treatment had roughly a 1.8‐fold increase in caspase‐3/7 compared to single‐agent treatment in SMS‐KCNR and SH‐SY5Y (Figure 5B). In contrast, BE(2)‐C and CHLA‐90 were shown to have no statistical difference in the level of caspase‐3/7 activation when treated with OTX015 alone or in combination with CGM097 (Figure 5B,C). The combination and OTX015 groups had roughly a 3.3‐ and 2.3‐fold increase in caspase‐3/7 expression compared to single‐agent CGM097 treatment in BE(2)‐C and CHLA‐90, respectively, after 32 hours of treatment (Figure 5B).

### 3.5 Treatment of NB xenograft mice with CGM097 and OTX015 in combination results in a significant delay in tumor growth and a significant increase in prolonging survival

**Figure 6:**
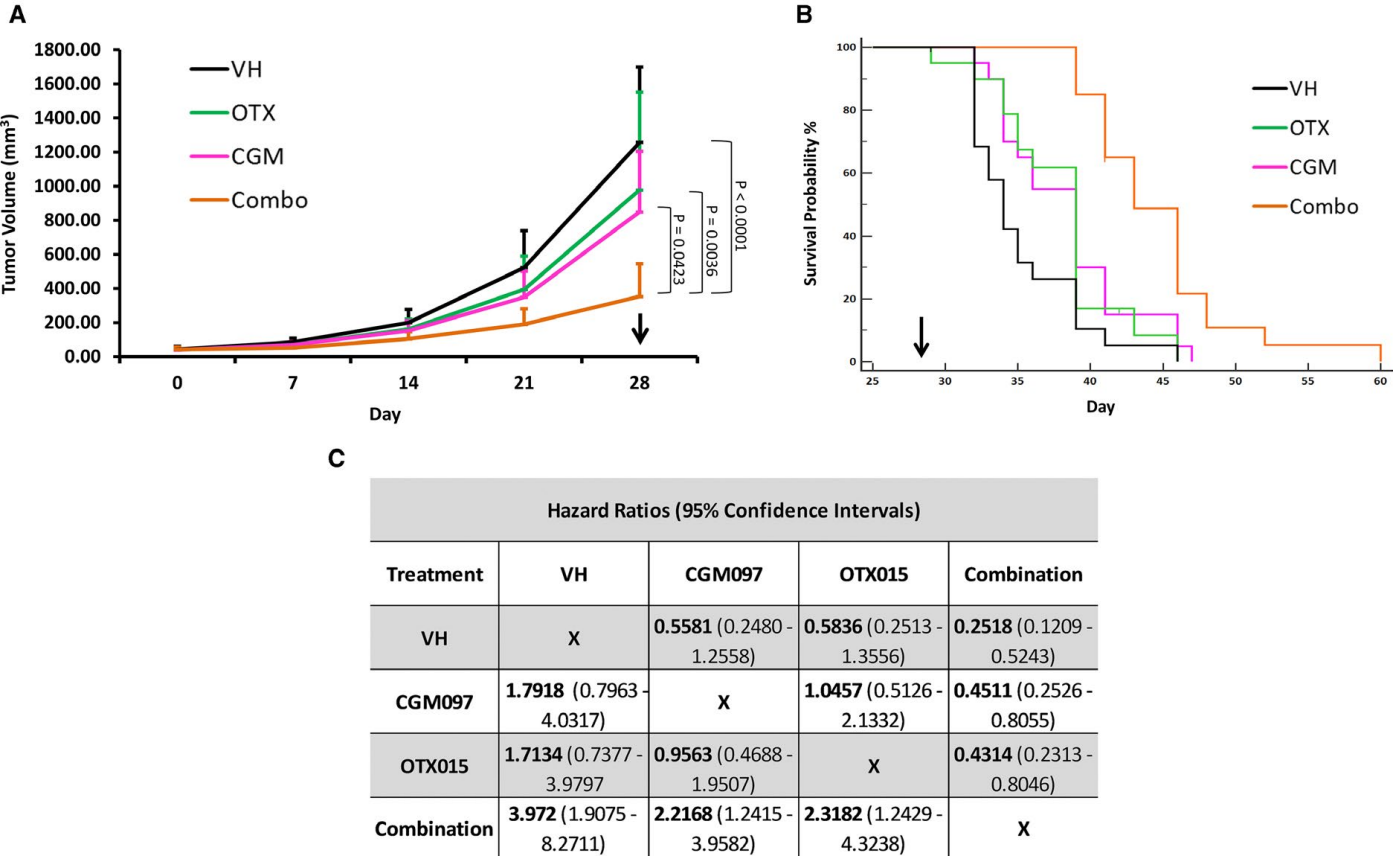
Combination treatment significantly reduces tumor volume and increases time‐to‐event. SMS‐KCNR xenograft mice were treated with vehicle (VH), 70 mg/kg CGM097, 50 mg/kg OTX015, or a combination of both drugs every day for 28 days. A, Tumor volume over time graph for SMS‐KCNR xenograft mice treated with vehicle, 70 mg/kg CGM097, 50 mg/kg OTX015, or a combination of both drugs. Data are represented as Mean ± SD. Data were analyzed by the Friedman test followed by Dunn's multiple comparison test. B, Kaplan‐Meier time‐to‐event graph for the mice corresponding to the tumor volume graph. Arrows represent end of treatment. C, Kaplan‐Meier plots were analyzed by a log‐rank test. The significance of the overall test was *P* < .0001 and the individual hazard ratios for each treatment group with their 95% confidence intervals are displayed. The experiment was conducted twice with 10 mice per group in each experiment

Our in vitro data led us to examine whether the combination of OTX015 and CGM097 is a viable co‐therapy in‐vivo. Toward this end, we utilized MYCN‐amplified SMS‐KCNR cells to model high‐risk disease that involves both increased MYC family expression and wild‐type p53 expression. SMS‐KCNR cells were subsequently injected into the right flank of nude mice. At the time of drug withdrawal (28 days after first treatment) the mice receiving the combination of CGM097 and OTX015 had an approximately 3.5 and 2.5 fold reduction in tumor volume compared to mice receiving vehicle or single‐agent alone, respectively (Figure 6A). The combination of drugs was also more effective in prolonging survival of the mice in these studies compared to single‐agent alone or vehicle (Figure 6B,C). Furthermore, there were no signs of weight loss or other toxicities in any treatment regimen given to the mice in these experiments (data not shown).

## 4 DISCUSSION

This study demonstrates that the combination of the MDM2 inhibitor CGM097 and the BET inhibitor OTX015 has the potential to be synergistically cytotoxic to NB cells expressing a wild‐type p53 protein. This is an important finding as CGM097 and OTX015 target the MDM2 and C‐MYC/MYCN pathways respectively, and these pathways result in an unfavorable prognosis in NB when they are highly active.^6^, ^32^ Some groups have utilized other BET inhibitors in NB, and it has been shown that the effects of BET inhibition are closely mimicked by shRNA knock down of MYCN, suggesting that this may be the mechanism by which OTX015 is cytotoxic to NB cells.^13^ Together, our data further implicate MDM2, MYC, and MYCN as viable drug targets in multiple NB cell lines.

Our data demonstrate that NB cells with a mutated p53 (CHLA90 and BE(2)‐C) are resistant to the MDM2 inhibitor CGM097, relative to wild‐type p53 cell lines (SMS‐KCNR and SH‐SY5Y), (Figure 2). Although a few EC50 values are below the line of Loewe additivity in the ray design analysis for BE(2)‐C and CHLA‐90, these are not considered statistically synergistic according to our analysis (Figure 2C). Additionally, the concentration of drug necessary to achieve synergy at those data points is substantially larger than those observed in SMS‐KCNR and SH‐SY5Y cell lines, which express a wild‐type p53. It is also plausible that the one statistically significant EC50 value in the CHLA‐90 cell line may be due to interactions of MDM2 with proteins other than p53, which has been reported.^33^ Conversely, SMS‐KCNR and SH‐SY5Y cells expressing a wild‐type p53 displayed a synergistic increase in cell death when treated with the combination of drugs (Figure 2C), suggesting that this observation may be at least in part due to p53 signaling.

Previous work has shown that NB cells with *MYCN* amplification are more sensitive to MDM2 inhibitors, potentially due to the known MDM2‐MYCN‐p53 interaction.^16^ Here we demonstrate that SH‐SY5Y, a cell line overexpressing *C‐MYC,* but with no *MYCN* expression, is sensitive to CGM097, suggesting that *MYCN* status may not be the only indicator of sensitivity of NB cells with wild‐type p53 to MDM2 inhibition (Figure 2A,C). It is worth noting that *C‐MYC* is not amplified in SH‐SY5Y cells but rather it is overexpressed due to a super‐enhancer translocation downstream of the *C‐MYC* transcriptional start site.^34^

**Figure 7:**
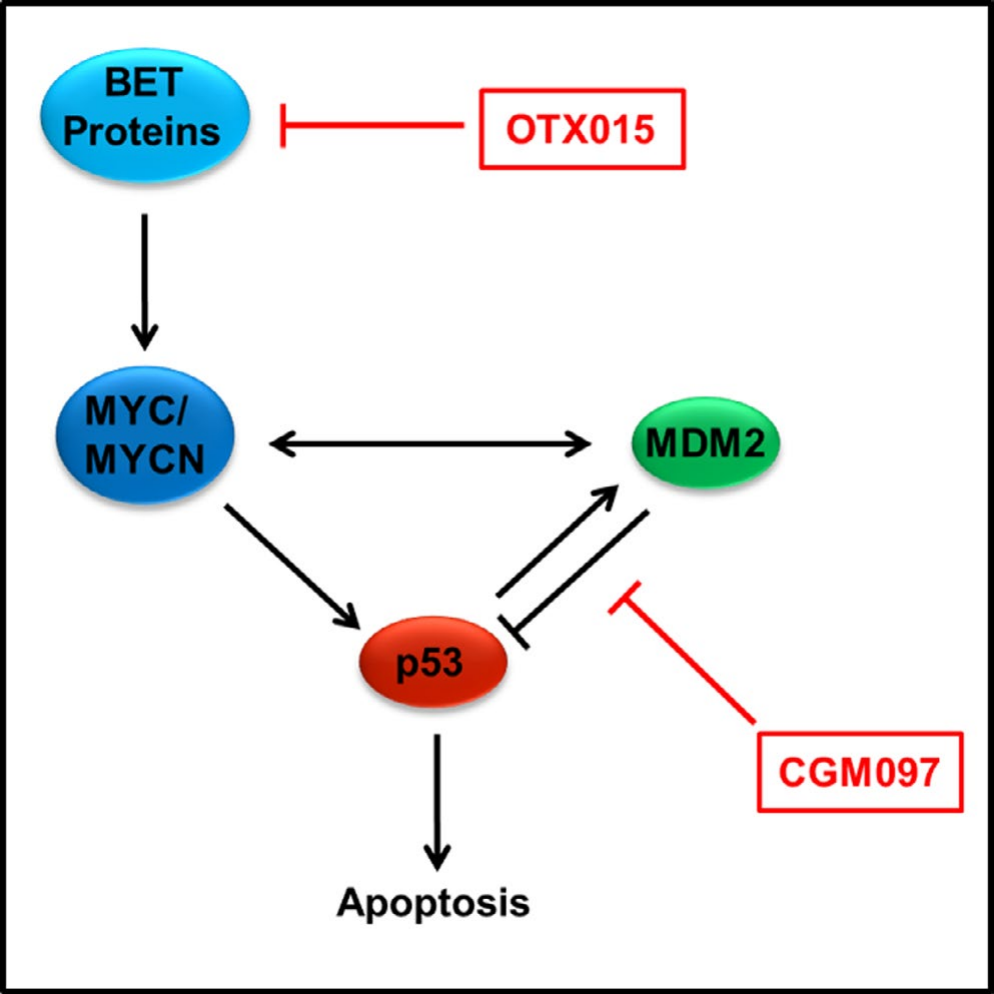
Hypothesized mechanism of action. Proposed mechanism of action for the combination treatment of CGM097 and OTX015 in neuroblastoma

p53 protein expression was significantly increased in the combination treatment in SMS‐KCNR compared to control, but not in SH‐SY5Y, the other p53 wild‐type cell line used in this manuscript (Figure 3B). This was surprising due to the similar apoptotic responses demonstrated in our IncuCyte experiments by both SMS‐KCNR and SH‐SY5Y cells when treated with the combination of CGM097 and OTX015 (Figure 5). Though the lack of p53 induction in combination treated SH‐SY5Y cells compared to single‐agent CGM097 treated cells is surprising, the level of p53 expression in the combination treated cells may be adequate to mediate its downstream effects. In single‐agent CGM097 treated SH‐SY5Y cells, the overexpression of MYC family proteins may alter the p53 response, as has been previously reported.^27^ MDM2 protein expression increased with CGM097 treatment in SMS‐KCNR and SH‐SY5Y, which is not entirely unexpected as increased p53 expression induces MDM2 in a negative feedback loop, which has been observed in the literature.^18^, ^19^, ^20^ This is further evidenced by the failure of CGM097 to elicit the same increase in MDM2 protein in the p53 mutant cells (Figure 3).^18^, ^19^, ^20^, ^21^ Additionally, single‐agent OTX015 treatment decreased the protein expression of MDM2 more than the combination treatment without a subsequent increase in p53 expression (Figure 3). This was unexpected as one would anticipate a decrease in MDM2 protein expression to correlate with an increase in p53 expression, as this follows the canonical pathway of p53 regulation. However, our RNA expression data demonstrate that MDM2 transcript is present at extremely high levels in these cell lines (Figure 1). Since basal MDM2 expression is at such high levels it is reasonable to hypothesize that there is still enough of the MDM2 protein after OTX015 treatment to maintain its inhibitory effect on p53 (Figures 1&3). Additional mechanistic studies will need to be performed to elucidate the exact means by which this combination is synergistic, as the interactions between these drugs may be more complicated than the mechanism we propose (Figure 7).

One functional endpoint of p53 activity is cell cycle arrest, often prior to the induction of apoptosis. Interestingly, only treatment with OTX015 or combination in SMS‐KCNR and SH‐SY5Y demonstrated a pronounced arrest in the transition from G1 to S phase in these cells compared to vehicle. This suggests that OTX015 can induce a cytostatic effect, while the p53 response in CGM097 primarily induces cell death in NB cells (Figures 4 and 5). It is reasonable to suggest that the downregulation of C‐MYC/MYCN may be responsible for the G1 arrest since MYC family proteins are potent mitogens and there was no induction of p21 or p53 with OTX015 treatment in the p53 wild‐type cell lines.^35^, ^36^ While p21 expression is generally associated with cell cycle arrest there is evidence of p21 induction leading to apoptosis in both p53‐dependent and ‐independent mechanisms.^37^ This could explain the lack of cell cycle arrest with CGM097 treatment despite the increase in p21 expression (Figures 3 and 5). Additionally, our analysis of cell cycle demonstrates a lack of cells in Sub G1, suggesting no decrease in viability, however, this experiment was conducted after 24 hours of drug treatment. While our IncuCyte data does suggest this is enough time to induce caspase‐3/7, it is potentially not enough time for the cells to complete the process of apoptosis and cleave genomic DNA (Figures 4 and 5).

BE(2)‐C and CHLA‐90 cells demonstrated a G1 cell cycle arrest and induction in apoptosis but only when treated with the BET inhibitor OTX015, and this effect was not altered by the addition of CGM097 (Figures 4 and 5). This effect was expected as these cells harbor a mutated p53 and are resistant to CGM097 at the concentrations utilized in this study. The G1 arrest in these cells could be due to the increase in p21 protein expression which must be induced by p53‐independent mechanisms, as demonstrated in other models (Figure 3).^38^ This p21 response in BE(2)‐C and CHLA‐90 would be in contrast to the p21 response we propose in SMS‐KCNR and SH‐SY5Y, which could be explained by the different mutation status of p53 in these cell lines (Figure 1). It is worth noting that while OTX015 and combination treatment did increase the protein expression of p21, this increase was not statistically significant (Figure 3B). Like CGM097, Be(2)‐C and CHLA‐90 are similarly more resistant to OTX015 when compared to SH‐SY5Y and SMS‐KCNR (Figure 2B). Although unexpected, this may be due to an increase in p21, which previous studies have shown to act as a suppressor of apoptosis in the absence of p53.^39^ Notably, the G1 arrest is more pronounced in SMS‐KCNR and BE(2)‐C compared to SH‐SY5Y and CHLA‐90 (Figure 4). This is most likely due to the high percentage of SH‐SY5Y and CHLA‐90 cells in the G1 phase at baseline compared to SMS‐KCNR and BE(2)‐C, which divide more rapidly (Figure 4).

Using nude mice bearing SMS‐KCNR xenografts, our *in viv*o approach further demonstrated that combination treatment with CGM097 and OTX015 potentiate an anti‐tumor effect. The mice receiving combination treatment not only had significantly smaller tumor volumes over the course of the studies but also had a pronounced increase in time‐to‐event (Figure 6A,B). These data demonstrate that the combination of the two drugs utilized in this study both slows tumor growth and prolongs life in vivo. Additionally, the mice receiving single‐agent or combination treatment experienced no adverse events or weight loss compared to the control group (data not shown). This suggests that the maximum tolerated doses for these drugs were not met in these studies, providing opportunity for a larger treatment effect with higher doses of these compounds. Even with the success of the combination in vivo, all the mice in the study eventually met the maximum tumor volume and had to be euthanized which could be due to using a sub‐maximal dose and removing the drug after 28 days of treatment.

The goal of the present study was to determine if the combination of the MDM2 inhibitor CGM097 and the BET inhibitor OTX015 could synergistically induce cell death in NB cell lines. Our data demonstrate that this drug combination is effective in NB cells with a functional p53. These data further support the importance of the roles that C‐MYC/MYCN and MDM2/p53 pathways play in NB. This combination of drugs may offer a new therapeutic approach to the treatment of NB patients, especially in high‐risk patients where *MYC* family oncogenes have been shown to have a greater role in disease. Although the current data suggest that the hypothesized mechanism may play at least a partial role in the described endpoints, it is largely limited by a need for further mechanistic studies. As such, additional experiments are needed to be to determine the mechanism by which CGM097 and OTX015 treatment results in synergistic cell death and prolonged survival in the models presented.

## Supporting information

Fig S1

Fig S2

## Data Availability

The data that supports the findings of this study are available from the corresponding author upon reasonable request.

## References

[1] Maris JM . Recent advances in neuroblastoma. N Engl J Med. 2010;362:2202‐2211. 20558371 10.1056/NEJMra0804577 PMC3306838

[2] Cheung NK , Dyer MA . Neuroblastoma: developmental biology, cancer genomics and immunotherapy. Nat Rev Cancer. 2013;13:397‐411. 23702928 10.1038/nrc3526 PMC4386662

[3] Strother D , van Hoff J , Rao PV , et al. Event‐free survival of children with biologically favourable neuroblastoma based on the degree of initial tumour resection: results from the Pediatric Oncology Group. Eur J Cancer. 1997;33:2121‐2125. 9516866 10.1016/s0959-8049(97)00293-1

[4] Kushner BH , Cheung NK , LaQuaglia MP , et al. Survival from locally invasive or widespread neuroblastoma without cytotoxic therapy. J Clin Oncol. 1996;14:373‐381. 8636746 10.1200/JCO.1996.14.2.373

[5] Nitschke R , Smith EI , Shochat S , et al. Localized neuroblastoma treated by a surgery: a Pediatric Oncology Group Study. J Clin Oncol. 1988;6:1271‐1279. 3411339 10.1200/JCO.1988.6.8.1271

[6] Brodeur GM , Castleberry RP . Neuroblastoma In: Pizzo PA, Poplack DG, eds. Principles and practices of pediatric oncology. Philadelphia: JB Lippincott; 1993:739‐767.

[7] Niemas‐Teshiba R , Matsuno R , Wang LL , et al. MYC‐family protein overexpression and prominent nucleolar formation represent prognostic indicators and potential therapeutic targets for aggressive high‐MKI neuroblastomas: a report from the Children’s Oncology Group. Oncotarget. 2018;9:6416‐6432. 29464082 10.18632/oncotarget.23740 PMC5814222

[8] Yang XH , Tang F , Shin J , Cunningham JM . A c‐Myc‐regulated stem cell‐like signature in high‐risk neuroblastoma: a systematic discovery (target neuroblastomas ESC‐like signature). Sci Rep. 2017;7:41. 28246384 10.1038/s41598-017-00122-x PMC5427913

[9] Lee DY , Hayes JJ , Pruss D , Wolffe AP . A positive role for histone acetylation in transcription factor to access to nucleosomal DNA. Cell. 1993;71:73‐84. 10.1016/0092-8674(93)90051-q 8422685

[10] Josling GA , Selvarajah SA , Petter M , Duffy MF . The role of bromodomain proteins in regulating gene expression. Genes (Basel). 2012;3:320‐343. 24704920 10.3390/genes3020320 PMC3899951

[11] Perez‐Salvia M , Esteller M . Bromodomain inhibitors and cancer therapy: from structures to applications. Epigenetics. 2017;12:323‐339. 27911230 10.1080/15592294.2016.1265710 PMC5453193

[12] Filippakopoulos P , Knapp S . Targeting bromodomains: epigenetic readers of lysine acetylation. Nat Rev Drug Discov. 2014;13:337‐356. 24751816 10.1038/nrd4286

[13] Puissant A , Frumm SM , Alexe G , et al. Targeting MYCN in neuroblastoma by BET bromodomain inhibition. Cancer Discov. 2013;3:308‐323. 23430699 10.1158/2159-8290.CD-12-0418 PMC3672953

[14] Delmore JE , Issa GC , Lemieux ME , Rahl PB , Shi J , Jacobs HM . BET bromodomain inhibition as a therapeutic strategy to target c‐Myc. Cell. 2011;146:904‐917. 21889194 10.1016/j.cell.2011.08.017 PMC3187920

[15] Noel JK , Iwata K , Ooike S , Sugahara K , Nakamura H , Daibata M .Development of the BET bromodomain inhibitor OTX015 [abstract]. In: Proceedings of the AACR‐NCI‐EORTC International Conference: Molecular Targets and Cancer Therapeutics; 2013 Oct 19–23; Boston, MA. Philadelphia (PA): AACR; Mol Cancer Ther 2013;12(11 Suppl): Abstract nr C244.

[16] Henssen A , Althoff K , Odersky A , et al. Targeting MYCN‐driven transcription by BET‐bromodomain inhibition. Clin Cancer Res. 2016;22:2470‐2481. 26631615 10.1158/1078-0432.CCR-15-1449

[17] Tweddle DA , Pearson ADJ , Haber M , et al. The p53 pathway and its inactivation in neuroblastoma. Cancer Lett. 2003;197:93‐98. 12880966 10.1016/s0304-3835(03)00088-0

[18] Carr‐Wilkinson J , O’Toole K , Wood KM , et al. High frequency of p53/MDM2/p14^ARF^ pathway abnormalities in relapsed neuroblastoma. Clin Cancer Res. 2010; 16: 1108‐1118. 20145180 10.1158/1078-0432.CCR-09-1865 PMC2842933

[19] Lu W , Pochampally R , Chen L , Traidej M , Wang Y , Chen J . Nuclear exclusion of p53 in a subset of tumors requires MDM2 function. Oncogene. 2000;19:232‐240. 10645001 10.1038/sj.onc.1203262

[20] Carr J , Bell E , Pearson AD , et al. Increased frequency of aberrations in the p53/MDM2/p14(ARF) pathway in neuroblastoma cell lines established at relapse. Cancer Res. 2006;66:2138‐2145. 16489014 10.1158/0008-5472.CAN-05-2623

[21] Van Maerken T , Speleman F , Vermeulen J , et al. Small‐molecule MDM2 antagonists as a new therapy concept for neuroblastoma. Cancer Res. 2006;66:9646‐9655. 17018622 10.1158/0008-5472.CAN-06-0792

[22] Weisberg E , Halilovic E , Cooke VG , et al. Inhibition of wild‐type p53‐expressing AML by the novel small molecule HDM2 inhibitor CGM097. Mol Cancer Ther. 2015;14:2249‐2259. 26206331 10.1158/1535-7163.MCT-15-0429 PMC4596780

[23] Slack A , Chen Z , Tonelli R , et al. The p53 regulatory gene MDM2 is a direct transcriptional target of MYCN in neuroblastoma. Proc Natl Acad Sci USA. 2005;102:731‐736. 15644444 10.1073/pnas.0405495102 PMC545522

[24] Gu L , Zhang H , He J , Li J , Huang M , Zhou M . MDM2 regulates MYCN mRNA stabilization and translation in human neuroblastoma cells. Oncogene. 2012;31:1342‐1353. 21822304 10.1038/onc.2011.343 PMC3213308

[25] Wang HQ , Halilovic E , Li X , et al. Combined ALK and MDM2 inhibition increases antitumor activity and overcomes resistance in human ALK mutant neuroblastoma cell lines and xenograft models. Elife. 2017;. 10.7554/eLife.17137 PMC5435462 28425916

[26] Barbieri E , Mehta P , Chen Z , et al. MDM2 inhibition sensitizes neuroblastoma to chemotherapy‐induced apoptotic cell death. Mol Cancer Ther. 2006;5:2358‐2365. 16985070 10.1158/1535-7163.MCT-06-0305

[27] Agarwal S , Milazzo G , Rajapakshe K , et al. MYCN acts as a direct co‐regulator of p53 in MYCN amplified neuroblastoma. Oncotarget. 2018;9:20323‐20338. 29755654 10.18632/oncotarget.24859 PMC5945521

[28] Gautier L , Cope L , Bolstad BM , Irizarry RA . affy – analysis of Affymetrix GeneChip data at the probe level. Bioinformatics. 2004;20:307‐315. 14960456 10.1093/bioinformatics/btg405

[29] Chou TC , Talalay P . Quantitative analysis of dose‐effect relationships: the combined effects of multiple drugs or enzyme inhibitors. Adv Enzyme Regul. 1984;22:27‐55. 6382953 10.1016/0065-2571(84)90007-4

[30] Belen'kii MS , Schinazi RF . Multiple drug effect analysis with confidence interval. Antiviral Res. 1994;1:1‐11. 10.1016/0166-3542(94)90089-2 7811057

[31] Lee JJ , Kong M . Confidence intervals of interaction index for assessing multiple drug interaction. Stat Biopharm Res. 2009;1:4‐17. 20037663 10.1198/sbr.2009.0001 PMC2796809

[32] Inomistova MV , Svergun NM , Khranovska NM , Skachkova OV , Gorbach OI , Klymnyuk GI . Prognostic significance of MDM2 gene expression in childhood neuroblastoma. Exp Oncol. 2015;37:111‐115. 26112937

[33] Bohlman S , Manfredi JJ . p53‐independent effects of MDM2. Subcell Biochem. 2014;85:235‐246. 25201198 10.1007/978-94-017-9211-0_13 PMC5507576

[34] Zimmerman MW , Liu Y , He S , et al. MYC drives a subset of high‐risk pediatric neuroblastomas and is activated through mechanisms including enhancer hijacker and focal enhancer amplification. Cancer Discov. 2018;8:320‐335. 29284669 10.1158/2159-8290.CD-17-0993 PMC5856009

[35] el‐Deiry WS , Tokino T , Velculescu VE . WAF1, a potential mediator of p53 tumor suppression. Cell. 1993;75:817‐825. 8242752 10.1016/0092-8674(93)90500-p

[36] Abbas T , Dutta A . p21 in cancer: intricate networks and multiple activities. Nat Rev Cancer. 2009;9:400‐414. 19440234 10.1038/nrc2657 PMC2722839

[37] Harper JW , Adami GR , Wei N , Keyomarsi K , Elledge SJ . (1993) The p21 Cdk‐interacting protein Cip1 is a potent inhibitor of G1 cyclin‐dependent kinases. Cell. 1993;75:806‐816. 10.1016/0092-8674(93)90499-g 8242751

[38] Jeong JH , Kang SS , Park KK , Chang HW , Magae J , Chang YC . p53‐independent induction of G1 arrest and p21 Waf1/Cip1 expression by ascofuranone, an isoprenoid antibiotic, through downregulation of c‐Myc. Mol Cancer Ther. 2010;7:2102‐2113. 10.1158/1535-7163.MCT-09-1159 20587660

[39] Karimian A , Ahmadi Y , Yousefi B . Multiple functions of p21 in cell cycle, apoptosis and transcriptional regulation after DNA damage. DNA Repair. 2016;42:63‐71. 27156098 10.1016/j.dnarep.2016.04.008

