# Supplementary material for: The MDM2 inhibitor CGM097 combined with the BET inhibitor OTX015 induces cell death and inhibits tumor growth in models of neuroblastoma
Source: Cancer Med. 2020 Oct 9;9(21):8144–58. doi: 10.1002/cam4.3407 (PMC7643634; doi:10.1002/cam4.3407)
Supplement: Supplementary file 1 — Fig S1 [file CAM4-9-8144-s001.pdf]

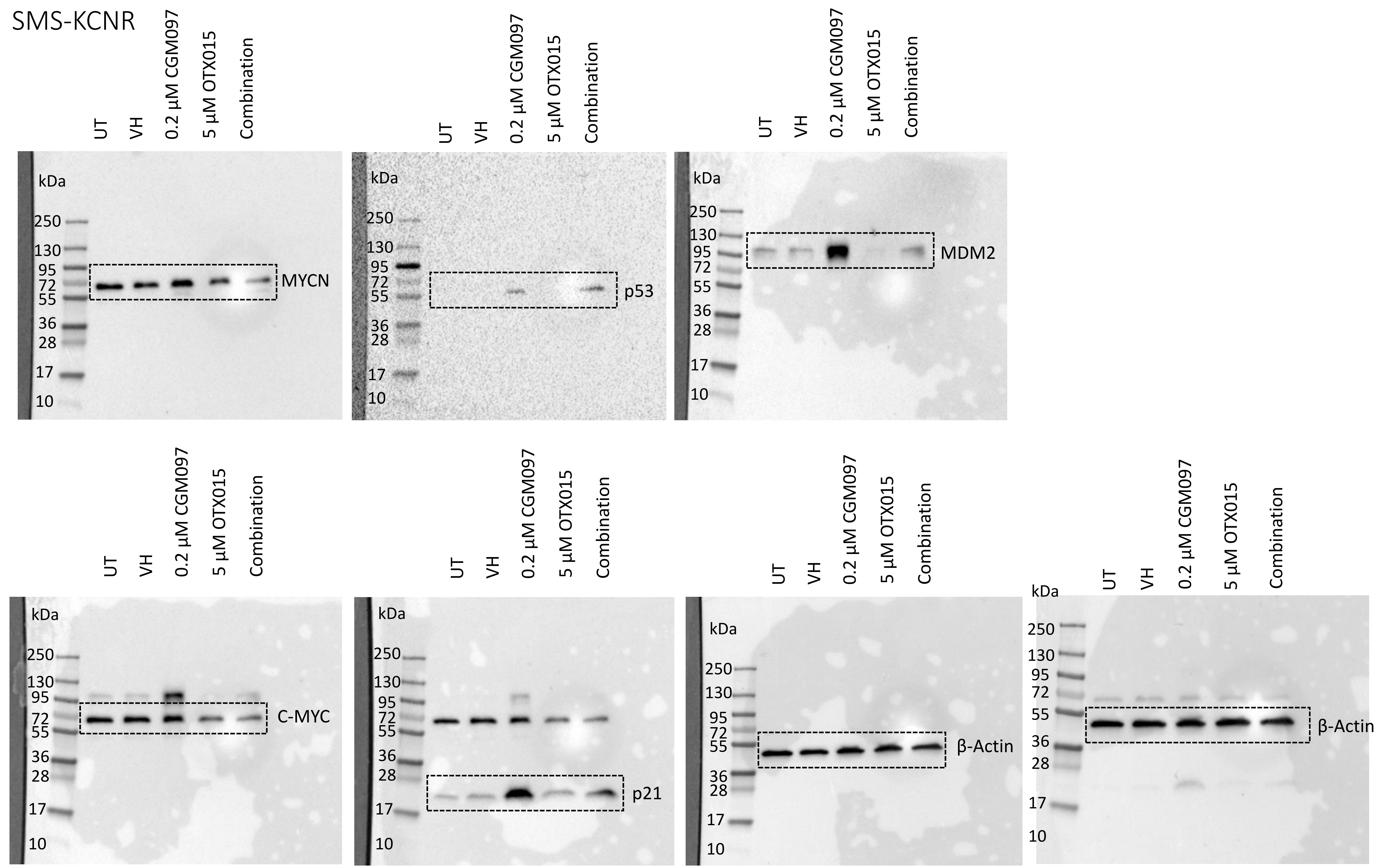
SMS-KCNR
UT
VH
0.2 µM CGM097
5 µM OTX015
Combination
kDa
250
130
95
72
55
36
28
17
10
MYCN
p53
MDM2
C-MYC
p21
β-Actin
β-Actin

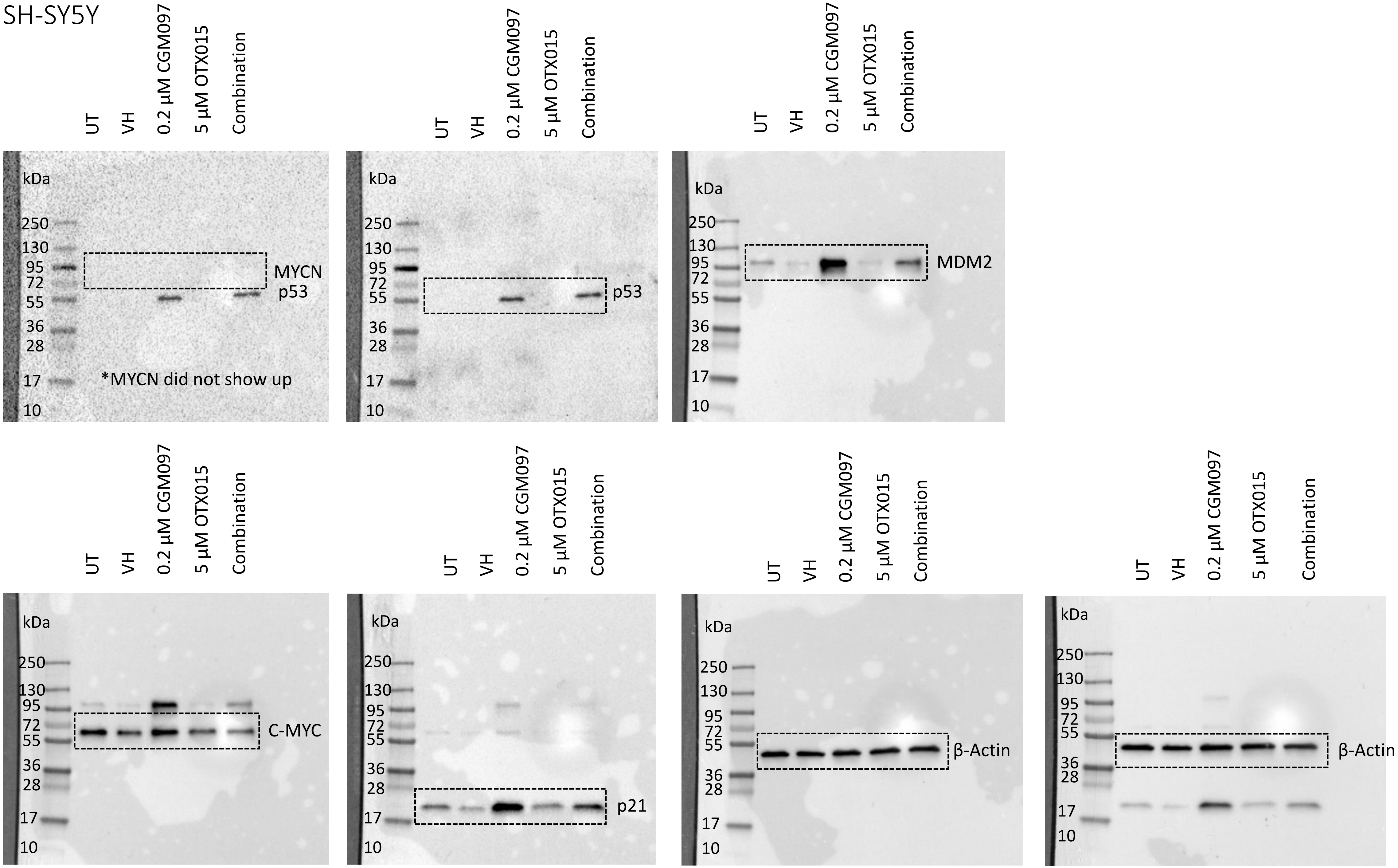
SH-SY5Y
UT
VH
0.2 µM CGM097
5 µM OTX015
Combination
kDa
250
130
95
72
55
36
28
17
10
MYCN
p53
*MYCN did not show up
MDM2
C-MYC
p21
β-Actin
β-Actin

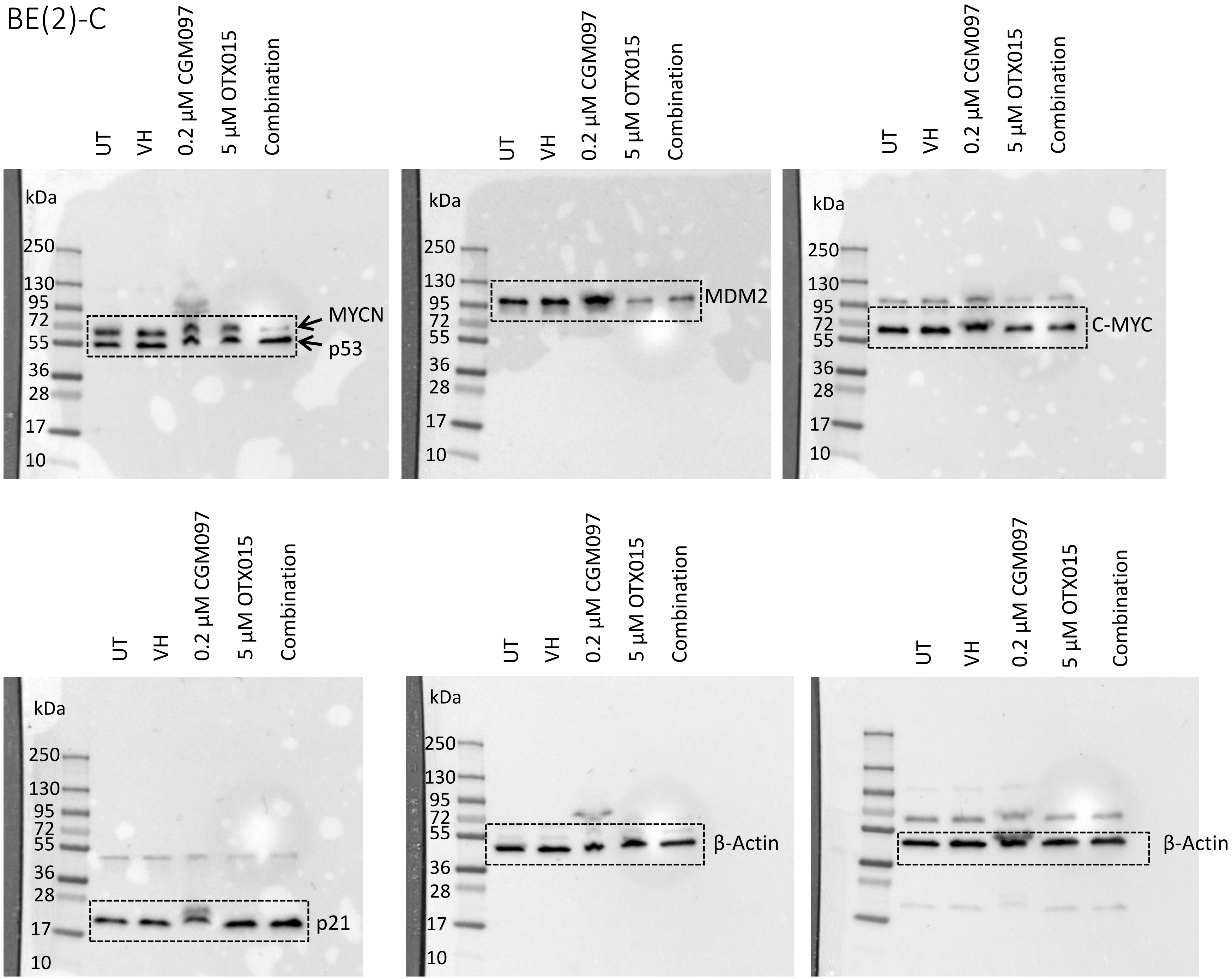

BE(2)-C
UT
VH
0.2 µM CGM097
5 µM OTX015
Combination
kDa
250
130
95
72
55
36
28
17
10
MYCN
p53
MDM2
C-MYC
p21
β-Actin
β-Actin

CHLA-90

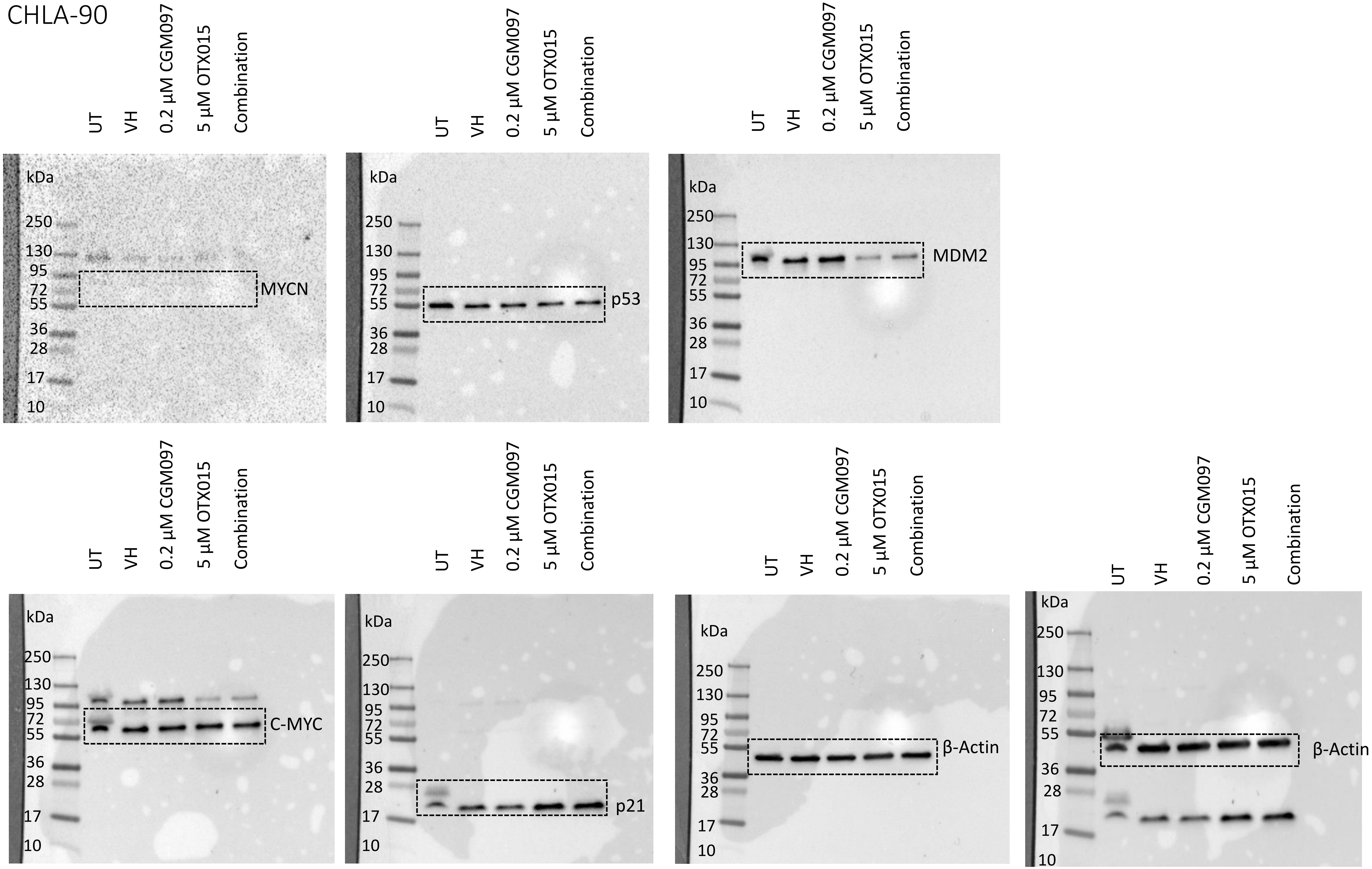
UT
VH
0.2 μM CGM097
5 μM OTX015
Combination
kDa
250
130
95
72
55
36
28
17
10
MYCN
p53
MDM2
C-MYC
p21
β-Actin
β-Actin
