# Supplementary material for: The MDM2 inhibitor CGM097 combined with the BET inhibitor OTX015 induces cell death and inhibits tumor growth in models of neuroblastoma
Source: Cancer Med. 2020 Oct 9;9(21):8144–58. doi: 10.1002/cam4.3407 (PMC7643634; doi:10.1002/cam4.3407)
Supplement: Supplementary file 2 — Fig S2 [file CAM4-9-8144-s002.pdf]

Supplemental Figure 2.

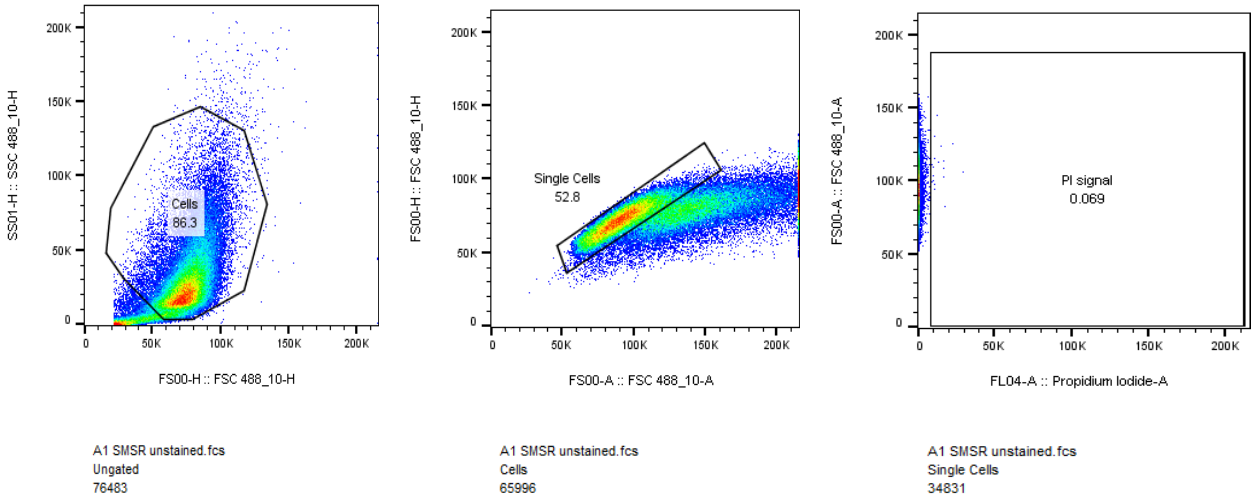

Gating strategy for the cell cycle analysis presented in Figure 4. The PI signal gate shown here was determined on unstained sample. The gating strategy presented is from a SMS-KCNR experiment and is representative of the gating strategy used across all cell lines.
